# Retrospective investigation of the 2019 African swine fever epidemic within smallholder pig farms in Oudomxay province, Lao PDR

**DOI:** 10.3389/fvets.2023.1277660

**Published:** 2023-09-29

**Authors:** Nina Matsumoto, Jarunee Siengsanan-Lamont, Tariq Halasa, James R. Young, Michael P. Ward, Bounlom Douangngeun, Watthana Theppangna, Syseng Khounsy, Jenny-Ann L. M. L. Toribio, Russell D. Bush, Stuart D. Blacksell

**Affiliations:** ^1^Sydney School of Veterinary Science, The University of Sydney, Camden, NSW, Australia; ^2^Mahidol-Oxford Tropical Medicine Research Unit, Faculty of Tropical Medicine, Mahidol University, Bangkok, Thailand; ^3^Section of Animal Welfare and Disease Control, Department of Veterinary and Animal Sciences, Faculty of Health and Medical Sciences, University of Copenhagen, Copenhagen, Denmark; ^4^National Animal Health Laboratory, Department of Livestock and Fisheries, Ministry of Agriculture and Forestry, Vientiane, Laos; ^5^Nuffield Department of Medicine, Centre for Tropical Medicine & Global Health, University of Oxford, Oxford, United Kingdom; ^6^Lao-Oxford-Mahosot Hospital-Wellcome Trust Research Unit (LOMWRU), Mahosot Hospital, Vientiane, Laos

**Keywords:** African swine fever, animal health economics, Lao PDR, pig production, smallholder, village, outbreak investigation

## Abstract

The 2019 African swine fever (ASF) outbreak in the Lao People’s Democratic Republic (Lao PDR or Laos) represented a major epidemiologic event where a transitioning lower-middle income nation (LMIC) experienced a viral epidemic in a naïve pig population. The diversity of pig management styles creates challenges for local and regional policymakers when formulating recommendations to control an ASF outbreak. The aim of this study were to investigate the management of pigs in villages of Oudomxay province that were affected by ASF in 2019, as a case study in a smallholder pig-rasing system in northern Laos. The frequencies of well known risk factors were measured in the affected villages and the timelines and household level stock losses due to the outbreak were investigated. These findings were compared to data available from a similar outbreak in the southern province of Savannakhet. Disease control implications of these findings are discussed. Mean losses were 3.0–23.3 pigs per household, with a mean lost herd value of USD 349, 95% CI (294–415). These pig losses reflect those estimated in Savannakhet (6.7 pigs per household). However, the financial loss estimated per household was higher, USD 349 versus USD 215, possibly due to higher pig values and a higher input/output management approach in Oudomxay. The investigation revealed the presence of numerous ASF risk factors, such as swill-feeding and free-ranging. In addition, poor biosecurity practices – such as inappropriate garbage disposal and slaughtering – that could contaminate the environment were present. ASF cases occurred across all villages between June and December 2019, with outbreak periods ranging from 22–103 days. These values are consistent with the outbreak in Savannakhet; however, notable differences in management styles were observed. These findings demonstrate the need for more disease control resources from the village to the Governmental level. Villages need support in enacting context appropriate biosecurity measures, whilst the ongoing surveillance and investigation of ASF require investment in logistical and veterinary resources at the Governmental level.

## 1. Introduction

The 2019 African swine fever (ASF) outbreak in the Lao People’s Democratic Republic (Lao PDR or Laos) remains a unique epidemiologic event where a transitioning lower-middle income nation (LMIC) experienced a viral epidemic in a naïve pig population. The first published data on the village-level impacts of this outbreak were collected in ASF-affected villages in the Southern province of Savannakhet (1).

Laos is a nation known for its diversity. While broadly classified as Lao-Tai, Hmong-Mein, Tibeto-Burman and Mon-Khmer, the Lao government recognises an additional forty-nine minority ethnic groups making up the Lao people, each with unique cultural practices, languages, and agricultural management styles (2, 3). There are distinct differences in the farming styles of those living in the Mekong floodplains and those in the mountainous regions that dominate the northern segment of the country (4). The diversity of pig management styles creates challenges for local and regional policymakers when formulating recommendations to control an ASF outbreak. For example, very few households reported the practice of swill-feeding their pigs in the Savannakhet ASF outbreak (1). Given this context, actions to prevent ASF might be adjusted to be more context appropriate. For example, the expenditure of minimal resources to prevent swill-feeding might be of lower priority when large amounts of foreign trader activity occur simultaneously (1).

In 2019, more than 150 confirmed ASF outbreaks occurred across Laos in just over six months (5). This followed the arrival of ASF in China from the Caucasus in 2018, with its subsequent spread to Vietnam, Cambodia in 2019 and more recently to Thailand (6). Due to the scale of the outbreak, an in-depth retrospective outbreak investigation to examine possible causative agents, the presence of known ASF risk factors, and impacts upon livelihoods was not possible. The risk factors for an outbreak are likely to vary between regions, requiring an investigation of ASF epidemic risk factors in a representative “northern” region. Based on the information generated, appropriate biosecurity recommendations can be made to prevent future ASF outbreaks by recognising the diversity of Laos’ pig farming communities.

The aim of this study was to investigate the management of pigs in villages of Oudomxay province (northern Laos) that were affected by ASF in 2019. The region was recommended by local animal health stakeholders as a good representation of the outbreak in northern Laos, in tandem with the work peformed in southern Laos. The frequencies of well known risk factors and other challenges to biosecurity in the management styles of the affected villages were assessed, and the timelines and household level stock losses due to the outbreak were investigated. In addition, these findings were compared to ASF data available from Savannakhet province (southern Laos). The disease control implications of these findings are discussed.

## 2. Materials and methods

### 2.1. Prior outbreak data

The Lao animal health services are governed by the Department of Livestock and Fisheries (DLF) of the Ministry of Agriculture, Forestry and Fisheries. At the village level, the Village Veterinary Worker (VVW) is a lay-person who has received a small amount of training from governmental and non-governmental organisations in basic veterinary care and diagnostics, they are the first person most villagers will consult upon illness in their animals (7). Upon the outbreak of ASF in the smallholder villages of Thapangtong district, the local VVW reported the unusual clinical signs and mortalities to their local District Agriculture and Fishery Office (DAFO), which in turn reported to the Provincial Agriculture and Fishery Office (PAFO) of the DLF (1). The initial outbreak investigations were performed by the DAFO/PAFO teams, who sent diagnostic samples and an outbreak report to the National Animal Health Laboratory (NAHL) and DLF, respectively. Upon confirmation, these already resource-poor teams returned to perform prevention and control activities such as culling, disinfection, movement controls and public awareness campaigns (1).

**Figure 1:**
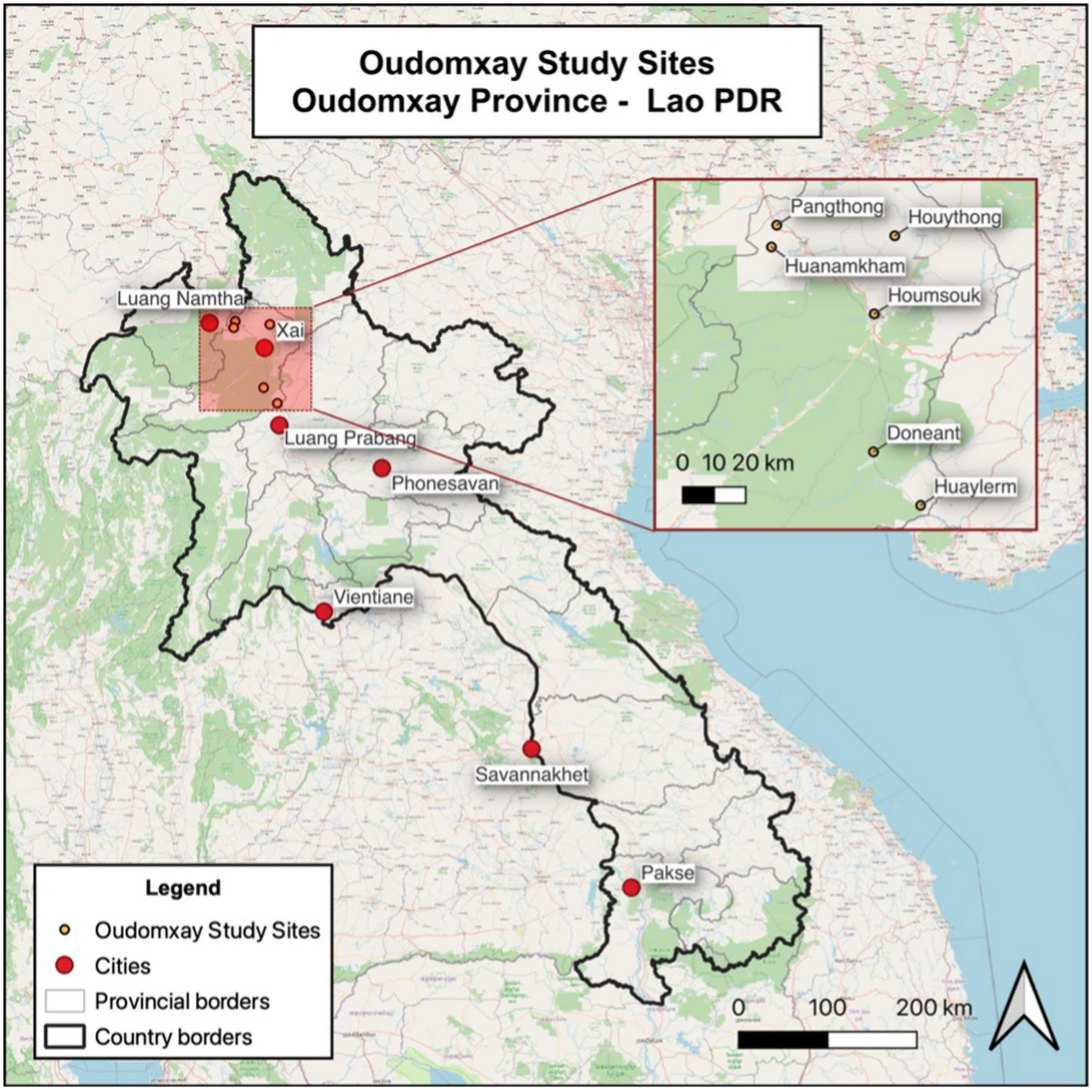
Map of Oudomxay province showing 2019 ASF outbreak locations; Huaycharng coordinates not available.

The villages included in this investigation were a census of all village-level outbreaks in Oudomxay province, where multiple households were affected (*n* = seven villages, official reporting data in Table 1). Oudomxay province was chosen purposively on the recommendation of the DLF both due to the high number of ASF cases and the availability of veterinary resources to perform questionnaires. All confirmed case villages tested positive Taqman rt-PCR for ASF on whole blood samples of clinically affected pigs at the Lao National Animal Health Laboratory (NAHL) (8). The locations of the villages where latitude and longitudinal data were available are shown in Figure 1. The location data was obtained from the 2011 Lao Agricultural Census and the DIVA-GIS Gazetteer (9).

**Table 1:** Morbidity and mortality data captured by the Oudomxay PAFO staff in the 2019 ASF outbreak.

Village	Affected households (total households)	Affected pigs (total pigs at risk)	Morbidity (household)	Mortality (pigs)	Deaths/affected HH^1^
Doneant	15 (101)	55 (101)	15%	54%	3.7
Homsouk	9 (93)	509 (725)	10%	70%	56.6
Houythong	19 (50)	144 (162)	38%	89%	7.6
Huanamkham	49 (80)	236 (273)	61%	86%	4.8
Huaycharng	12 (68)	223 (316)	18%	71%	18.6
Huaylerm	58 (101)	167 (169)	57%	99%	2.9
Pangthong	12 (68)	32 (181)	18%	18%	2.7
Total	174 (561)	1,366 (1927)	31%	71%	7.9

^1^HH – household.

The data collected by the Oudomxay PAFO at the time of the initial outbreak investigation in 2019 are presented in Table 1, based on a central spreadsheet (Microsoft Excel) kept by the Oudomxay PAFO team. Upon their arrival at an ASF suspected village, local investigators sampled a small number of pigs showing ASF-like clinical signs. However, the number of pigs and the households sampled were not recorded. The team recorded the total number of pig-owning households in the village, the number of pigs at risk, the number of households affected (household morbidity) and the number of pigs that had already died (mortality).

After an initial investigation, the PAFO team sent samples to the NAHL for testing and a report to the DLF, which are recorded in Table 2. The DLF Found Date was obtained from the PAFO records, the NAHL report date and diagnosis date were collated from the NAHL’s Pathogen Asset Control System, a diagnostic sample inventory database.

**Table 2:** Reporting dates from the DLF and NAHL for villages affected by the 2019 ASF outbreak in Oudomxay, Laos.

Village	DLF found date	NAHL report date	NAHL diagnosis
Doneant	8/08/2019	4/08/2019	6/08/2019
Homsouk	19/07/2019	18/07/2019	19/07/2019
Houythong	1/08/2019	1/08/2019	1/08/2019
Huanamkham	16/08/2019	29/08/2019	30/08/2019
Huaycharng	20/06/2019	–	–
Huaylerm	29/07/2019	28/07/2019	28/07/2019
Pangthong	2/08/2019	3/08/2019	3/08/2019

### 2.2. Sampling methodology

The study period was defined as 1 June 2019 to 1 January 2020. A case village was a village with PCR confirmation of ASF in porcine blood samples during the study period based on the NAHL diagnosis date. A case household was defined as any household where pigs displayed signs of ASF, as defined in Sánchez-Vizcaíno et al. (10), and a case animal displayed clinical signs of ASF. Ethics approval for the questionnaires was obtained from the University of Sydney Human Ethics Committee under approval number (2019/725).

Where villages had less than twenty-five households affected by ASF, the investigators aimed to perform as close to a household-level census as possible. In the case of a village with more than twenty-five ASF-affected households, the same methods were used as those in Matsumoto et al. (1). The maximum number of interviews was determined for simplicity of instruction to the field teams and ability to complete surveys within the allocated days. The Village Chief or VVW drew up a list of all available ASF-affected households. A random number generator (Microsoft Excel) was used to select a list of twenty-five households. In both scenarios, some households were not available on the days of the questionnaire and replacements could not be found, and they were not included in the investigation. Furthermore, some ASF-affected households fell outside of the study period or clinical signs consistent with ASF were not observed. Of the 161 participant households, 108 met the case definition for ASF-affected in this study, and only their data is presented in the outbreak investigation section of this paper. However, the data from all 161 households are still presented for informative purposes relating to the biosecurity and management of pigs in the affected villages.

### 2.3. Questionnaire design and administration

The questionnaire comprised twenty-eight open and closed questions on the households’ herd size, structure, price value, management styles (encompassing feeding, housing, and health practices), trading history prior to the outbreak and disease history during the outbreak period. Questions were designed based upon literature review of risk factors for ASF, with a focus on gaining relevant epidemiologic data for an outbreak investigation. A detailed explanation of the questionnaire design process, which included a round of pilot testing prior to the final questionnaires performed in Savannakhet province in 2019, and a copy of the survey can be found in Matsumoto et al. (1, 11).

The data presented here is at the household level; however, the epidemic curves present the total number of pig mortalities per day since there was notable variation in the herd sizes between households and therefore the number of daily mortalities.

Travel restrictions within Laos substantially delayed the delivery of the questionnaires during the SARS-CoV 2019 pandemic; as such, all questionnaires were completed in December 2020. The questionnaire delivery otherwise mirrored that of Matsumoto et al. (1). The questionnaires were performed in the Lao language by DLF officers from PAFO and DAFO. After the questionnaire, each participant was given an educational t-shirt about ASF in pigs to recognise the donation of their time.

### 2.4. Data handling and analysis

All questionnaires were translated from Lao to English and entered into Microsoft Excel by a team of Lao veterinarians working at NAHL. The data were then exported into RStudio for cleaning, descriptive analyses, financial analysis and outbreak investigation (12).

Financial losses were calculated using the questionnaire data, by summing the farmer reported value of the pigs lost during the outbreak. For example if they reported owning one sow worth 800,000 Lao kip (LAK) and two piglets worth 100,000 LAK each, the lost herd value was 1,000,000 LAK. The value was then converted into 2019 USD for reporting (8814.07 LAK to 1 USD). This was then reported as the lost herd value.

## 3. Results

### 3.1. Pig management and biosecurity practices prior to the outbreak

#### 3.1.1. Overall herd demography

Across the seven villages, there was considerable variation in the herd sizes of households. For this reason, the average herd demography is presented here by herd size from large (more than nine pigs) to very small (less than three pigs) (Table 3). The sizes demonstrated here were chosen based on the previous data in Savannakhet where households tended to own a sow with one to six of her piglets (1). Larger and medium herds tended to include more fattening pigs and have more piglets per sow, while smaller herds had equivalent numbers or more sows than piglets. Very few households owned a boar.

**Table 3:** Pig herd demography of ASF-affected villages in Oudomxay, Laos.

Herd size (total no. pigs)	HH^1^	Average herd size (pigs)	Piglets	Fatteners	Sows	Boar
Large (>9)	33	16.4	12.1	1.5	1.9	0.9
Medium (7–9)	38	7.9	4.5	1.1	1.6	0.7
Small (3–6)	53	4.3	1.7	1.0	1.3	0.4
Very small (<3)	37	1.4	0.2	0.6	0.7	0.0

^1^HH – households.

#### 3.1.2. Housing styles

The questionnaire participants typically kept their pigs in household-based pens (72.7%). Another 14.9% kept their pigs in some form of free-ranging, including free-ranging during the day and penning them at night. Other housing methods included multi-household pens, tethering the pigs in the garden, or keeping the pigs in the crawl space under the house (Table 4). It was common for farmers to provide multiple housing methods from the list of responses (i.e., free-range AND tethered) for their pigs, likely reflecting seasonal changes in housing styles reported by Okello et al. (13).

**Table 4:** Housing styles in ASF-affected villages of Oudomxay, Laos.

Housing type	*N*	%	Average weekly cost (USD)
Penned – communal pen	20	12.4	11.55
Free-range	24	14.9	11.77
Other*	31	19.3	25.77
Penned – own pigs	117	72.7	11.34
No answer	1	0.6	0

*For respondants that did not identify their pig housing style as penned or free-range.

#### 3.1.3. Feeding and water sources

The participants reported feeding commercial brand diets (19.9%), local vegetable products (15.5%) or a mixture of the commercial and vegetable diets (14.3%). To determine the frequency of pig exposure to potentially infective meat, smallholders were asked “Do you feed swill to your pigs?” with the follow up question “If yes do you cook it beforehand?,” as well as “Do you feed household scraps to your pigs.” Swill feeding (1.9%) and household scrap feeding (37.3%) were both reported. An additional ten households (6.2%) did not respond to the direct question of swill feeding their pigs, but then reported cooking the swill that they fed to their pigs, and another three reported that they did not cook the swill or were not sure (1.9%).

Commercial diets such as that of Charoen Pokphand Foods and Thai and Vietnamese brands were reported as common pig feeds, often combined rather than brand exclusive. Local vegetable products mentioned included rice and rice bran, maize, cassava, pumpkins, and banana tree flowers/stems. The most common primary water source was the local river (22.8%) or a communal well in the village (21.0%). It was unclear in the data if the pigs could freely access these water sources or if the water was transported to the pigs’ enclosures.

#### 3.1.4. Local slaughtering practices

Across the study population, many households did not routinely slaughter their own pigs (40.4%). Of the households that reported home slaughtering of their stock for food or ceremonial purposes, the majority (39.8%) slaughtered one to three pigs annually in an area outside the home (Table 5). Lao regulations state that all slaughter should occur at an official slaughterpoint, and home slaughter can only occur for ceremonial reasons and under the supervision of the VC and VVW (14).

**Table 5:** Home slaughtering locations of ASF-affected villages in Oudomxay, Laos.

Butchering location	*N*	%
Home – inside area of the home	56	34.8
Home – outside area of the home	78	48.5
Village – dedicated area	1	0.6
No answer	26	16.2

Qualitative information on the utilisation of home-killed pork demonstrated that most tissues (blood, skin, viscera, bones and offal) were kept for food. A small number of questionnaire respondents reported leaving blood or viscera (1.6% and 0.8%, respectively) on the ground. The practice of feeding pork bones to the dogs in the village was relatively common (31.6%).

#### 3.1.5. Trading activities

Four smallholder pig trading activities were recorded during the study period in Doneant, Huaycharng and Huaylerm villages. However, smallholder participants also reported numerous trading activities outside of the study period, ranging November 2018 – November 2020 (thirty purchases, two sales and thirteen who reported a date of trading without providing further information). Only three were reported from local traders; the rest were either transactions between neighbours or no answer was given. The trader purchases all occurred in Homsouk village in 2020. The average number of pigs bought in a transaction was 4.8, and the average number sold was 3.5. Prior to trading, pig owners are required to complete a number of health, vaccination and ownership transfer certificates – however further questioning on this topic was outside the scope of the survey (14).

#### 3.1.6. Pig contact structures

Some of the surveyed smallholders in Oudomxay reported their pigs had contact with their neighbours’ pigs and with wild boar daily (4% and 8%, respectively). However an additional 92% provided no answers for the question on their neighbours’ pigs, and 93% did not answer the question on wild boar.

#### 3.1.7. Disease management

VVWs attended 5.0% of households during the study period. None of the VVW visits occurred on the same days as one another. Smallholder farmers reported a wide range of common therapeutic agents for pigs, such as penicillin–streptomycin and oxytetracycline. Five farms (3.1%) performed routine piglet prophylactic care, such as iron and vitamin injections. Households spent an annual average of USD 10.59 on medications and USD 3.02 on vaccines for their pigs.

In the event of a disease outbreak, the questionnaire participants were asked to describe how they disposed of the carcasses of disease-affected animals. Most participants buried their dead pigs at depths ranging from less than one metre to greater than two metres, with the most common depth being one to one-and-a-half metres (91.8%). Other disposal methods included burning the carcasses (2.0%) or retaining the carcass for consumption (0.7%).

### 3.2. 2019 African swine fever outbreak

#### 3.2.1. DLF outbreak data

The Oudomxay PAFO of the DLF provided the outbreak data they collected during the ASF epidemic, including data on the numbers of affected pigs and households (Tables 1, 2). The DLF reported finding the ASF-affected villages 20 June 2019–16 August 2019, whilst the NAHL received the reports of these cases 18 July 2019–29 August 2019; in some cases, the dates of case detection by the DLF were after receipt of an official report at the NAHL (*n* = three of six villages with NAHL report dates). Huaycharng village had no dates recorded and no explanation was provided. There is no centralised database to corroborate these dates.

The DLF provided population data for the study. In the study villages, 10%–61% of all households and 18%–99% of all pigs were clinically affected when the DLF commenced control measures (Tables 1, 2).

#### 3.2.2. Questionnaire household outbreak data

The number of questionnaire participants affected by ASF and their herd sizes are shown in Table 6. Of the 161 participants, 108 fit the study case definition for households and pigs, and their data are presented here. The mean losses of pigs per household ranged from three to twenty-three mortalities, and the mean value of the lost herds across all households was USD 349, 95% CI [294, 415]. In Homsouk, Huaycharng and Pangthong, more survey participants fit the study’s case definition for an ASF-affected household than were recorded in the original DLF data.

**Table 6:** Retrospective outbreak investigation questionnaire mortality data from households affected by the 2019 ASF outbreak in Oudomxay, Laos.

Village	No. participant HH^a^	Mortalities (no. pigs)	Mean losses/HH^a^ (SD)	DLF reported cases (*n*, %)
Doneant	14	42	3 (2.4)	15 (93%)
Homsouk	10	233	23.3 (16.3)	9 (111%)^b^
Houythong	12	62	5.2 (2.9)	19 (63%)
Huanamkham	25	153	6.1 (2.8)	49 (51%)
Huaycharng	17	125	7.4 (4)	12 (142%)^b^
Huaylerm	14	60	4.3 (2.6)	58 (24%)
Pangthong	16	121	7.6 (3.8)	12 (133%)^b^
Overall	108	796	7.4 (7.7)	174 (62%)

a HH – household.

b Villages where more households fit the study case definition than reported in the DLF data.

**Figure 2:**
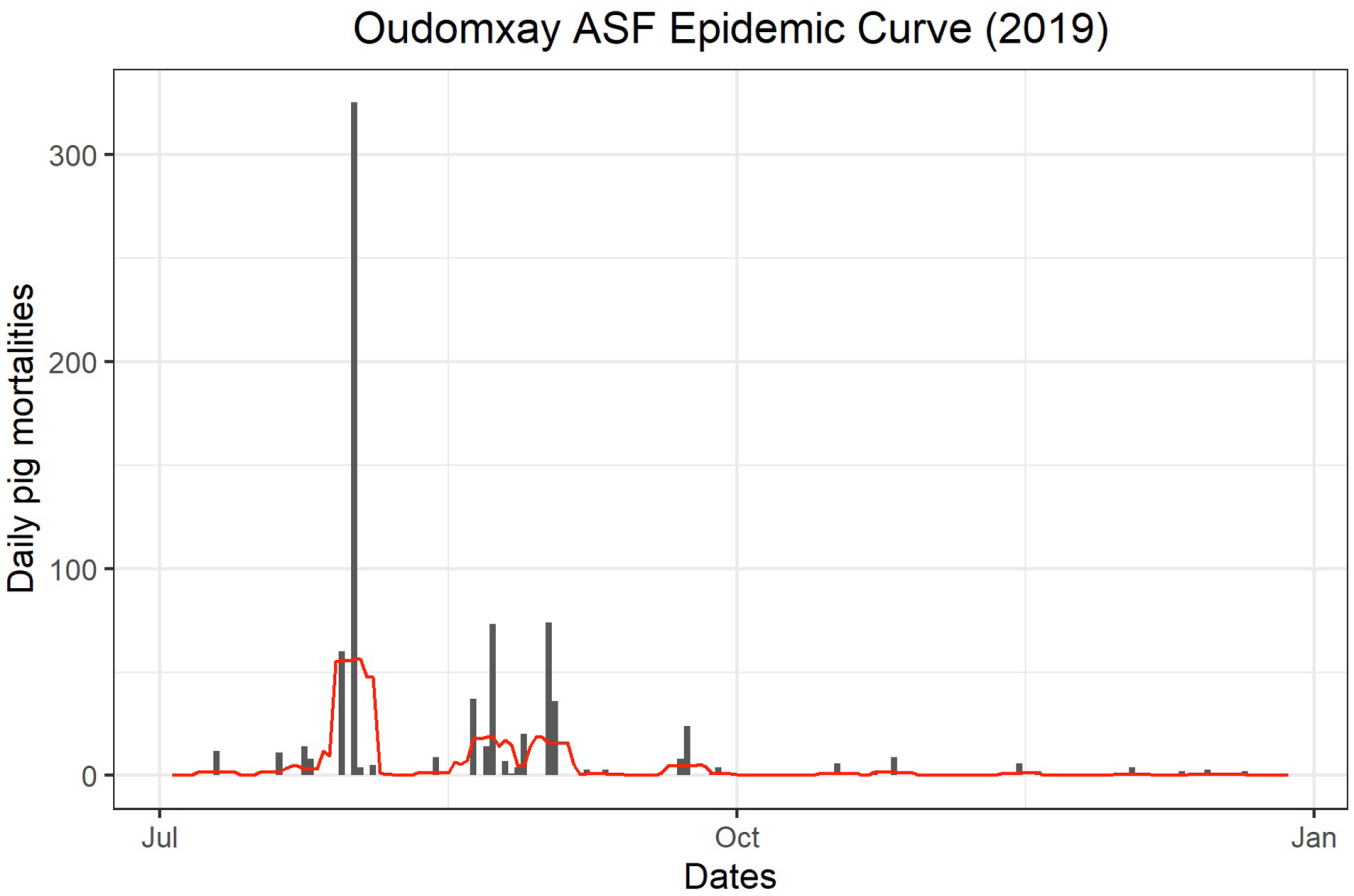
Epidemic curve of owner-reported pig mortalities during the 2019 ASF outbreak in Oudomxay, Laos.

The first reported mortalities consistent with ASF clinical signs occurred in July 2019 in Panthong village (Table 7). The epidemic curve (Figure 2) shows that peaks in mortalities occurred between July and October of 2019, with sporadic mortalities continuing through December 2019. The villages where the first reported mortality occurred on 1 August may not represent an accurate date, as the first date of the week or month was recorded if the participants could not remember an exact date (Table 7).

**Table 7:** Retrospective timeline data from households affected by the 2019 ASF outbreak in Oudomxay, Laos.

Village	Case dates			Outbreak period (days, IQR)
	First	Median	Last	
Doneant	22/09/2019	7/11/2019	21/12/2019	90 (48)
Homsouk	1/08/2019^1^	1/08/2019^1^	2/09/2019	32 (23)
Houythong	1/08/2019^1^	1/08/2019^1^	1/08/2019^1^	–
Huanamkham	1/08/2019^1^	20/08/2019	23/08/2019	22 (9)
Huaycharng	1/08/2019^1^	1/08/2019^1^	23/09/2019	53
Huaylerm	22/08/2019	2/09/2019	3/12/2019	103 (27)
Pangthong	10/07/2019	30/07/2019	4/08/2019	25 (5)
Overall	10/07/2019	1/08/2019	21/12/2019	164 (27)

^1^ Household could not remember dates of cases.

#### 3.2.3. Clinical presentation

**Figure 3:**
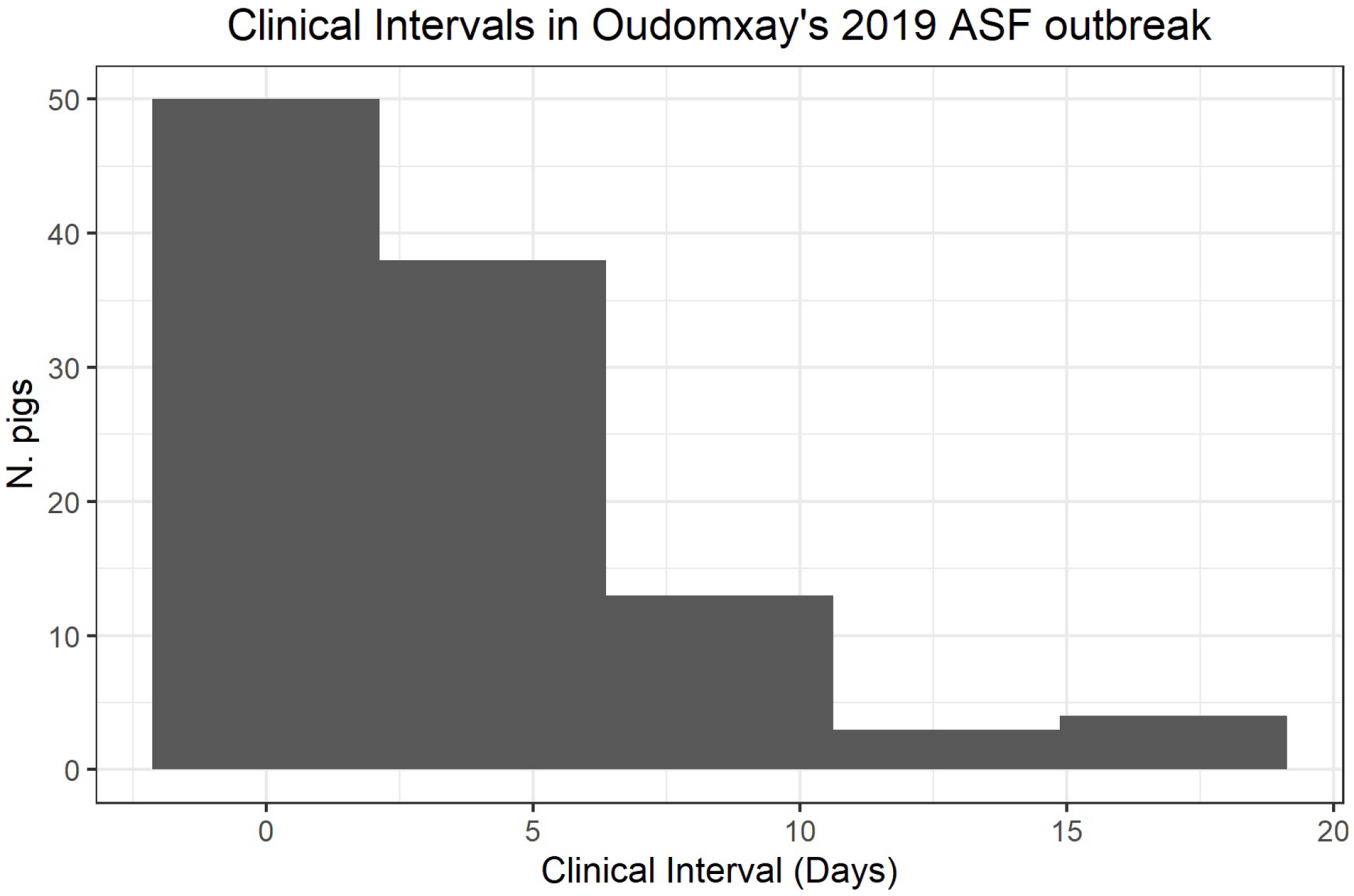
Intervals from the appearance of clinical signs to mortalities in pigs affected by the 2019 ASF outbreak in Oudomxay, Laos.

The average owner-reported interval between the onset of clinical signs and mortality was 3.6 days (IQR five days, Figure 3). Fifty-three households reported the clinical interval to be two days or less, suggestive of a peracute outbreak, whilst sixty-eight households reported clinical periods of three days or more, suggestive of more acute syndromes.

The questionnaire participants were asked to describe their affected pigs’ early and late clinical signs. The most common early clinical signs reported were weakness (29.9%), sudden death (32.0%) and anorexia (25.9%). The most common late clinical signs were death (40.7%) and reddened body or reddened spots on the body (15.3% and 6.8%, respectively). Other late clinical signs observed included conjunctivitis, fever, salivation, tremors, and reddened papillae.

## 4. Discussion

### 4.1. Outbreak investigation

The outbreak periods where mortalities were occurring based on the questionnaire were 22–103 days, likely reflecting the variations in both number of households affected per village (nine to fifty-eight households in the DLF data) and the number of affected pigs across the villages (32–509 pigs in the DLF data). In addition to population size, differences in management styles, such as the prevalence of free-ranging or swill-feeding, likely influenced the speed of spread between households and, therefore, the variation in outbreak lengths. The outbreak periods in the smaller Oudomxay villages were similar to those of Savannakhet, with inter-quartile ranges of 5.5–35 days (1). NAHL reports and diagnosis dates, DLF dates and questionnaire response timelines varied across the villages. The DLF reported the outbreaks between 20 June and 16 August 2019, whilst the first mortalities with consistent clinical signs occurred between 10 July 2019 and 22 August 2019. A centralised disease reporting database and future work investigating the period of whole-village infectivity will aid in clarifying the dates and periods of outbreaks under investigation, and future work should aim to compare the risk factors between villages within the study.

The number of pig mortalities per household was similar in the Lao DLF and the questionnaire data, with discrepancies likely caused by recall bias and random error related to the specific participating households in larger villages. Mean losses were 3.0–23.3 pigs per household, with a mean lost herd value of USD 349, 95% CI [294, 415]. These pig losses are consistent with those of Savannakhet (6.7 pigs per household). However, the financial loss estimated in Savannakhet (USD 215 per household) is notably different (1). The variation in management styles between the two locations may be a plausible explanation for the differences in herd loss value. A full gross margin analysis would aid comparison of financial losses caused by the outbreaks at both locations, as feed inputs and health inputs were also higher in Oudomxay households than in Savannakhet households before the outbreak.

In managing the ASF outbreak, the Lao DLF performed culling and disinfection activities at the whole village level upon PCR confirmation of ASF. The NAHL lacked the resources to perform concurrent testing for similar clinical syndromes. The case definition for ASF in the individual pig was based on clinical signs rather than molecular diagnostics in this study. ASF clinical signs are notoriously non-specific, particularly in peracute and acute cases (10). It is possible that other similar diseases, such as Classical Swine Fever or Highly-Pathogenic Porcine Respiratory and Reproductive Syndrome Virus, were present and causing concurrent mortalities at the same time as the ASF outbreak. The study period was limited to June–December 2019 to reduce overestimating mortalities caused by similar diseases, to align with the first reported incursion of ASF into Laos in June 2019 (5). Ongoing active surveillance activities and abattoir-based surveillance would aid in better understanding the background disease load on smallholder pig farmers impacted by an ASF outbreak.

An additional and unexpected source of error in this study was the SARS-CoV-19 pandemic. The pandemic forced deployment of the questionnaires to be delayed by over 12 months. This long delay created difficulties for the farmers in remembering exact case numbers, clinical signs, and dates of events. Some of this recall may also be due to education levels or access to accurate data recording tools within smallholder villages. A centralised reporting system combined with lifetime traceability on animals would allow for more accurate timelines and calculations of stock losses in future outbreaks.

Additionally, training and resources for VVWs to record mortality or morbidity events in their communities would allow for cross-referencing by the local veterinary authorities in future studies. Further household training could include animal health management records, with disease events, mortalities and medication/vaccination administrations recorded in a simple wall calendar or similar. This could further be strengthened with production records to include reproductive outcomes such as farrowing dates and trading records for historical reference.

### 4.2. Investigation of management and risks to biosecurity

This study aimed to investigate management that could increase disease transmission risk through direct and indirect contacts, such as free-ranging or trader activities. Whilst only 1.9% of farmers reported feeding swill to their pigs, 37.3% reported feeding household scraps. An additional 6.2% reported cooking the swill, but did not report feeding swill. The confusion around these responses suggest that questions around feeding practices need to be clearly defined, given feeding household scraps *is* swill feeding. Slaughter products left out or improperly disinfected create a source of ASF environmental contamination. All tissues and secretions from the ASF-affected pig are infectious (15–17). The blood of a viraemic pig is extremely contagious, and ASFV contained in infected faeces can survive in the environment for up to 3.7–8.5 days, depending on the ambient temperature (15–17). Environmental contamination is of particular concern in smallholder settings where farmers may attempt to salvage pork meat products from their slaughtered or suddenly deceased animals. Many farmers (31.6%) reported giving pork bones to their dogs after slaughter, allowing parts of an infectious carcass to be spread well beyond the initial slaughter site and across the village. In scenarios where free-ranging occurs, the behaviour of domestic free-range pigs likely mirrors that of wild hogs, where they remain in family groups but come into contact at common resources (18, 19).

The use of common water sources noted in this study must be investigated further as a potential cause of spread. This complex interplay warrants close observation to understand all possible risk factors within the smallholder village. Supporting local veterinary workers in their biosecurity and consulting skills would provide a regionally relevant pathway to understanding the biosecurity challenges of smallholder pig farming.

The pig-raising styles in Oudomxay was notably different from Savannakhet prior to the 2019 ASF outbreak. Oudomxay households tended to keep more pigs in higher-density settings, with more money invested in feeding, medicating, and housing their pigs. Herd sizes were larger and of higher value than the previously surveyed ASF-affected households in Savannakhet. The increased value per pig is likely due to the higher value feed and medical inputs utilised amongst the Oudomxay participants, such as commercial brand diets and routine piglet care. Adding more nutrient-dense commercial feeds and vegetable crops to the diet of a village pig can improve both the growth rate of the piglet and the reproductive performance of a sow (20). In addition, routine piglet health care, such as iron injections and vitamin supplementation, is well reported to improve piglet survivability (21). In the large herds, the piglet-to-sow ratio was approximately six piglets per sow (1). Whilst not comparable to benchmarks for commercial operations, this is dramatically higher than the three piglets per sow reported in Savannakhet. Both estimates are comparable to similar studies in non-outbreak conditions. In a study of small farms (less than 30 sows) in the northern provinces of Xayaburi and Phongsaly, households reported an average of 7–7.2 piglets born alive and 4.3–6.2 piglets weaned per sow (22). The variation in value and herd sizes may be due to management styles or a more general shift in Lao farming styles reflecting the broader pattern of economic growth in the region. Ongoing assessment of both management and value chains can help pork stakeholders better understand the changing needs of their industry as Lao progresses beyond the status of a least-developed country. The reported low number of boar ownership would also warrant further investigation, as renting or borrowing boars, or practicing artificial insemination could also represent an ASF transmission risk factor, despite its lack of regulation in Lao PDR (14).

The medical protocols described in the questionnaire are of interest beyond investigating an ASF outbreak. Participant households reported using common veterinary antibiotics such as oxytetracycline and penicillin–streptomycin, with the majority reporting their use for weakness or fever as a two-to-three day course. Antibiotics are not recommended for treating ASF because it is a viral disease. Future studies into antimicrobial use amongst smallholders would be of value, as one smallholder reported using weekly oxytetracycline and vitamin injections on their fattening pigs. Prophylactic antimicrobial usage can effectively reduce the load of common respiratory pathogens in pigs and grow larger marketable animals. Oral antibiotics are common in commercial operations (23, 24). However, the routine use of antimicrobials on-farm significantly increases the risk of antimicrobial resistance (AMR) (25). Smallholder farming systems lack the resources to manage the emergence of AMR; in Timor-Leste, a study of smallholders found that only 12.7% understood what antibiotics were, and even fewer knew their mode of action (26). The utilisation of antimicrobials amongst Lao smallholders must be further studied to protect the future of smallholder pig health and welfare.

Trader activity during an outbreak provides numerous possibilities for anthropogenic ASF spread between villages (27). The emergency sale of sick pigs is a well-documented behaviour among smallholder farmers (28, 29). Most recorded trading activities occurred between neighbours within close geographical proximity, with some trader activities occurring after the study period. The lack of detail on the identities of trading partners and often the quantities of animals sold may reflect cultural attitudes towards the Government and reporting of income. Furthermore, a lack of resources to accurately track or record trading data may have contributed to this problem. In Xayaburi province, a social network analysis demonstrated that most trader-smallholder interactions occurred in a discrete network, with a small number of traders servicing a specific region (30). A social network analysis of smallholder and trader activity in the region would allow investigators to understand the dynamics of trading interactions as a potential route for disease transmission. This behaviour may vary between ethnic groups and geographical regions.

The presence of VVW in smallholder villages allows nations with under-resourced veterinary services to provide baseline animal health and welfare services but simultaneously increase the risk of disease transmission if not managed correctly (31). The Lao DLF report provides training to the local VVWs when resources become available (7). In ASF outbreaks, there have been occurrences where veterinarians and para-veterinarians inadvertently become mechanical fomite vectors as they travelled between ASF-affected sites without suitably disinfecting their tools and equipment (32). Recent work in neighbouring Cambodia demonstrated gaps in para-veterinary service biosecurity (33). Savannakhet VVWs reported washing their syringes and needles in soap and water rather than complete disinfection or using new needles and syringes between animals, which would not allow for sufficient disinfection of ASFV (1). Eight households recorded a VVW visit during the outbreak period, which may have allowed for ASF transmission between herds. Ongoing training and support for LMIC paraveterinary and veterinary services will support smallholder animal health and welfare whilst reducing iatrogenic infection risk.

The purposive nature of performing the study in Oudomxay province introduces a source of selection bias which must be accounted for when extrapolating the findings to larger ASF modelling projects and decision-making. The selection bias may therefore over- or under-represent the management and outbreak data findings. The regions selected in this study and the work of Matsumoto et al. (1) were nominated by the Lao DLF as representative regions for northern and southern Laos due to their accessibility and the availability of on-ground veterinary resources. For this reason, both studies may represent a best-case scenario for pig-rearing and outbreak management. Despite this potential bias, many of the descriptive results align with previous work on pig-rearing in Lao smallholder villages in other provinces like Savannakhet, Luang Prabang, Phongsaly and Xayaburi (1, 3, 22, 34). Ongoing research into the impact of the ASF outbreak on smallholder farming across various Lao contexts is necessary.

This study identified numerous transmission pathways by which ASF could spread within – and between villages in an outbreak. Housing, slaughter, wild-boar contacts, feeding, and watering protocols observed in this study allow for effective contact between infectious and susceptible pigs, whilst trading and para-veterinary activities could hasten the spread of the disease between whole villages. The presence of wild boar in Laos has been confirmed *in camera* trap studies between 2013 and 2017, however the distribution, ecology and nature of interactions with domestic pigs are poorly understood, and this study therefore presents a rare piece of information suggesting that contact occasionally occurs between these groups (35, 36). Regarding the frequency of risk factor events, it appears that between-village activities occur less frequently and may be a more resource-efficient method of controlling disease spread. Within the village, further research is necessary to understand the best methods for reducing household-to-household spread. An initial pilot performed in Timor-Leste found that combining public awareness campaigns with simple, community-driven biosecurity strategies such as fencing and reduced free-ranging and cleaning measures appeared to reduce the incidence of ASF (37). Potential focus areas for Lao smallholders may include these approaches and adapted methods for household garbage disposal and feeding practices to optimise biosecurity.

Smallholder farming at its best is a regenerative system in the cyclical nature of inputs and outputs, with minimal waste and highly efficient utilisation of all resources. Using pig manure as a fertiliser for crops is beneficial and a potential source of contamination in an outbreak. Almost all farmers reported the consumption of all pig products, including offal, an important practice for a region where almost a third of all children are stunted in their growth due to low protein (38). Traditional outbreak questionnaire studies such as this may fail to capture the nuances of such a system. In non-outbreak scenarios, a more complex understanding of Lao smallholder agriculture may be developed using methods such as system dynamics and spatial group model building, as has been piloted in Timor-Leste (39). Future studies into developing a smallholder biosecurity assessment tool that is sensitive to this style of farming and cultural practices would allow local animal health staff and outreach organisations to teach smallholder pig farmers good biosecurity practices whilst efficiently maintaining their outputs.

The investigation of the 2019 ASF outbreaks in Oudomxay province showed that practices recognised as risk factors for ASF were present among the 7 villages, such as swill-feeding and free-ranging. In addition, poor biosecurity practices, such as inappropriate garbage disposal and slaughtering that would contaminate the environment, were present. These findings demonstrate the need for increased resources from the village to the Governmental level. Villages need support in enacting context-appropriate biosecurity measures, whilst the ongoing surveillance and investigation of ASF require investment into logistical and veterinary resources at the Governmental level. The findings of this research provides outlines for future work in supporting smallholder farming in rural areas both within and beyond the South East Asian context.

## Data availability statement

The raw data supporting the conclusions of this article will be made available by the authors, without undue reservation.

## Ethics statement

The studies involving humans were approved by University of Sydney Human Ethics Committee under approval number (2019/725). The studies were conducted in accordance with the local legislation and institutional requirements. The participants provided their written informed consent to participate in this study.

## Author contributions

NM: Formal analysis, Investigation, Writing – original draft, Writing – review & editing. JS-L: Project administration, Writing – review & editing. TH: Formal analysis, Methodology, Writing – review & editing. JY: Conceptualization, Supervision, Writing – review & editing. MW: Supervision, Writing – review & editing. BD: Writing – review & editing. WT: Project administration, Writing – review & editing. SK: Project administration, Writing – review & editing. J-AT: Supervision, Writing – review & editing. RB: Supervision, Writing – review & editing. SB: Funding acquisition, Project administration, Supervision, Writing – review & editing.

## Funding

The author(s) declare financial support was received for the research, authorship, and/or publication of this article. This research was partially funded by the Biological Threat Reduction Program (BTRP) of the Defense Threat Reduction Agency (DTRA) of the US Government (contract number HDTRA1-08-D-0007). SB is supported by the Wellcome Trust (220211) of the United Kingdom.

## Conflict of interest

The authors declare that the research was conducted in the absence of any commercial or financial relationships that could be construed as a potential conflict of interest.

The author(s) declared that they were an editorial board member of Frontiers, at the time of submission. This had no impact on the peer review process and the final decision.

## Publisher’s note

All claims expressed in this article are solely those of the authors and do not necessarily represent those of their affiliated organizations, or those of the publisher, the editors and the reviewers. Any product that may be evaluated in this article, or claim that may be made by its manufacturer, is not guaranteed or endorsed by the publisher.
